# Hydrogen-producing small intestinal bacterial overgrowth is associated with hepatic encephalopathy and liver function

**DOI:** 10.1371/journal.pone.0264459

**Published:** 2022-02-25

**Authors:** Kunihiko Yokoyama, Akira Sakamaki, Kazuya Takahashi, Takumi Naruse, Chihiro Sato, Yuzo Kawata, Kentaro Tominaga, Hiroyuki Abe, Hiroki Sato, Atsunori Tsuchiya, Kenya Kamimura, Masaaki Takamura, Junji Yokoyama, Shuji Terai

**Affiliations:** 1 Division of Gastroenterology and Hepatology, Graduate School of Medical and Dental Sciences, Niigata University, Niigata, Japan; 2 Department of General Medicine, School of Medicine, Niigata University, Niigata, Japan; Nihon University School of Medicine, JAPAN

## Abstract

Overt hepatic encephalopathy (HE) is one of the complications of liver cirrhosis (LC), which negatively affects the prognosis and quality of life of patients. Small intestinal bacterial overgrowth (SIBO) is significantly associated with LC and its complications, including HE. We investigated the relationship between SIBO and LC, and the difference between hydrogen-producing and methane-producing SIBO (H-SIBO and M-SIBO, respectively). This is a prospective cohort study of 107 cases. Breath measurements of hydrogen and methane concentrations were performed for the diagnosis of SIBO. The study cohort included 81 males with a median age of 70 (40–86) years, and SIBO was detected in 31 cases (29.0%). There were no significant differences between the SIBO positive and SIBO negative groups. Reclassification into H-SIBO (16 cases) and others (91 cases) was performed, and the Child-Pugh score was only derived in the multivariate logistic analysis (P = 0.028, odds ratio 1.39, 95% confidence interval 1.04–1.85). Furthermore, H-SIBO was significantly associated with covert HE in chi-square test (50.0% vs. 24.2%, P = 0.034). In addition, we evaluated the therapeutic response on SIBO of rifaximin in eight covert HE patients. 20% patients with M-SIBO and 67% patients with H-SIBO showed an improvement of the breath test. In conclusion, H-SIBO, but not M-SIBO, is significantly associated with liver function, and rifaximin might be more effective for covert HE with H-SIBO. Therefore, the diagnosis of SIBO, including the classification as H-SIBO and M-SIBO, might help to determine the choice of treatment for HE.

## Introduction

Liver cirrhosis (LC) is the end stage of chronic hepatitis regardless of any etiology. D’Amico et al. reported that the 5-year survival rate of the natural course of LC is approximately 75% in compensated LC and only approximately 25% in decompensated LC [1]. Hepatic encephalopathy (HE) is a neuropsychiatric syndrome characterized by an impaired consciousness complicated by acute or chronic severe liver dysfunction [2], which negatively affects the prognosis and quality of life; the 1-year survival is 73% and the 3-year survival is 38% [3, 4]. The prevalence of overt HE is 10%–14% in patients with LC [5, 6]. and overt HE has a preliminary stage called covert HE, which could be difficult to diagnose because of its mild symptoms or no symptoms at all. Therefore, neuropsychiatric tests are often used for the diagnosis of covert HE [7]. In addition, mild cognitive impairment and psychomotor deficit due to covert HE are associated with patients’ health-related quality of life [8], and patients with covert HE advanced to overt HE at high rates [9]; therefore, diagnosis and effective therapeutic intervention at the stage of covert HE may lead to a better outcome.

Meanwhile, the concept of small intestinal bacterial overgrowth (SIBO) has recently attracted attention. SIBO is characterized by abnormal bacterial overgrowth in the small intestine, which is associated with mucosal inflammation and malabsorption [10]. SIBO can be associated with various diseases, such as irritable bowel syndrome (IBS) and chronic pancreatitis. Cirrhosis is also known to be associated with SIBO [11]. The frequency of SIBO in cirrhotic patients was reported by a meta-analysis to be 34.8%–47.1%, with an odds ratio of 6.83 against non-cirrhotic patients; in addition, SIBO was significantly associated with several LC complications, such as covert HE, ascites, and spontaneous bacterial peritonitis [12].

Although there is no gold standard for the diagnosis of SIBO, two diagnostic methods have been reported. First, proximal jejunal fluid cultivation was used as the direct method for the diagnosis of SIBO; however, that method has several limitations: the invasiveness of the proximal jejunum fluid collection, the presence of nonculturable bacteria (approximately 60% of the intestinal microbiota) [13], and the possibility of oral bacterial contamination. Next, the breath test was used as the indirect method. The mechanism of the test is that orally ingested sugar substrates are metabolized by gut bacteria, and their metabolites are detected in the breath. Because the breath test is easy to perform and is minimally invasive, it is used for the detection of SIBO worldwide [12]. The diagnosis of SIBO using the breath test can be classified into the hydrogen-producing type and methane-producing type. The guideline of the American college of gastroenterology proposed the name “intestinal methanogen overgrowth” (IMO) instead of “methane-producing type SIBO” because methanogens are not bacteria but archaea, and that overgrowth of archaea is not limited to the small intestine but extends to the large intestine [14].

Herein, we reported the relationship between SIBO diagnosed using the breath test and liver function tests and the incidence rate of covert HE in patients with LC. We also investigated the difference between hydrogen-producing SIBO (H-SIBO) and methane-producing SIBO (M-SIBO) because the distributions and pathogeneses of H-SIBO and M-SIBO are considered to be very different.

## Materials and methods

### Inclusion and exclusion criteria

**Fig 1:**
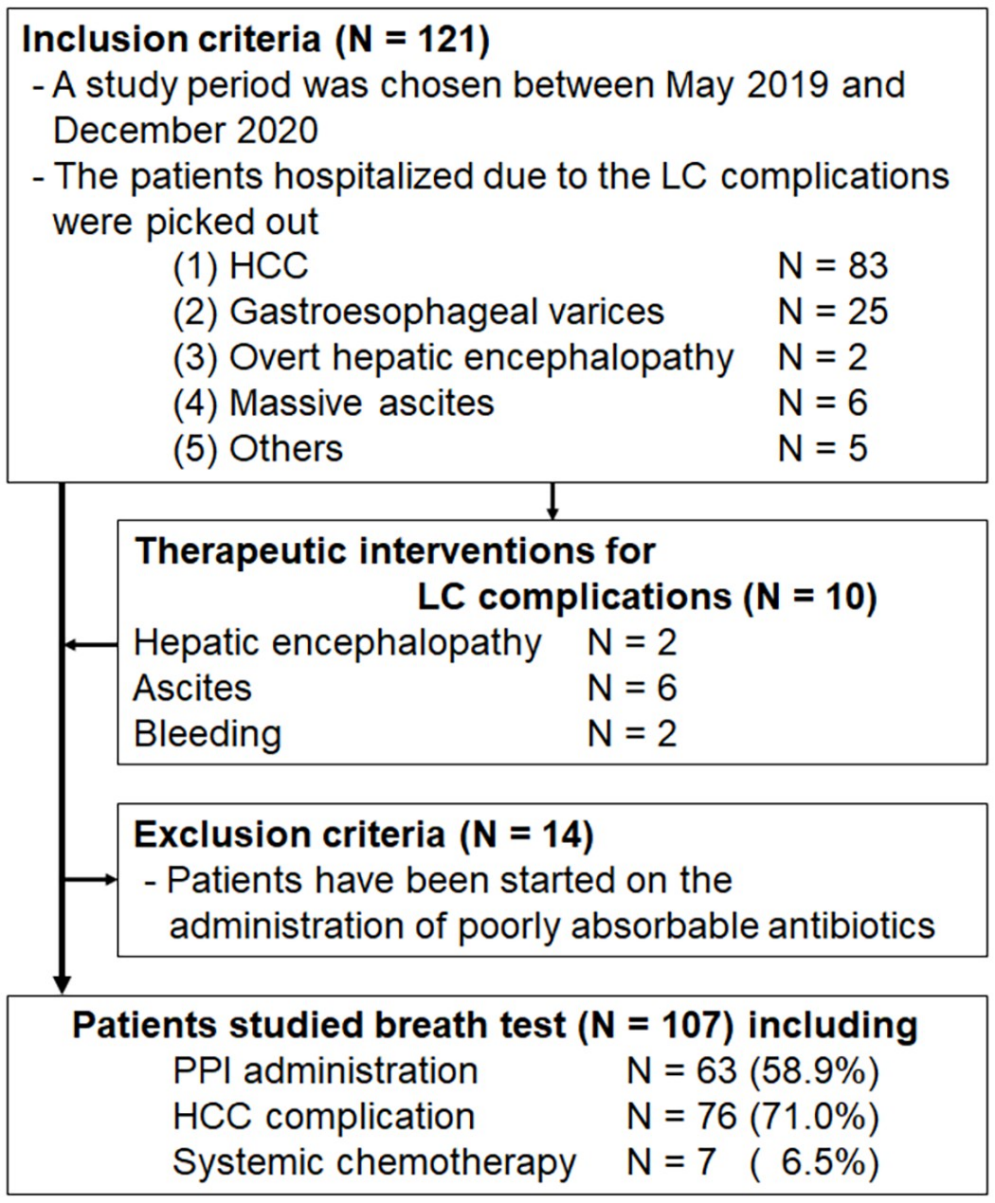
Patient selection procedure and study procedure. LC, liver cirrhosis; HCC, hepatocellular carcinoma; PPI, proton pump inhibitor.

This was a cross-sectional and prospective cohort study carried out in Niigata University. The study was approved by the ethical review board of Niigata University (Approval Number 2019–0226). We picked out the patients hospitalized because of LC complications, such as hepatocellular carcinoma (HCC) (83 cases), gastroesophageal varices (25 cases), overt HE (2 cases), ascites (6 cases) and others (5 cases), between May 2019 and December 2020, and 121 patients were included the study. All patients were agreed with the study concept and provided their written informed consent. The breath test was performed after the management of LC complications that require acute treatment, such as bleeding, overt HE, and ascites. However, in patients intended for elective treatment, such as HCC and gastroesophageal varices, breath test was performed before the treatment. HCC complication was defined as the presence of clinically viable HCC with the imaging study and serum tumor markers at the time of the breath test. In addition, 14 patients were excluded because of rifaximin administration, poorly absorbable antibiotics, at the time of the breath test; so we analyzed 107 cases in the cross-sectional study. No patients were received any other antibiotics. The authors will ensure that this study is conducted in accordance with the principles of the Declaration of Helsinki and with the ethical guidelines for medical and biological research involving human subjects in Japan. The patient selection procedure and study procedure are presented in Fig 1.

### Diagnosis of SIBO

The breath test was used for diagnosing SIBO in the study. For an accurate diagnosis, bread, pasta, and noodles, which increase hydrogen production, were not allowed for 24 h prior to the breath test. In addition, oral intake was not allowed for 12 h before the test without water and tea. Regular medication was taken 2 h before the test, if necessary, and drinking, exercise, and smoking were prohibited after that. Oral rinsing was to be performed to avoid the metabolism of sugar substrates by oral bacteria.

First, breath measurement was performed three times before the sugar substrate loading, and the average score was used as the baseline. Next, breath measurement was performed every 15 min after loading and up to 120 or 180 min. Fifty grams of glucose or 10 g of lactulose was used as the sugar substrate; lactulose was considered in patients with poorly controlled diabetes (seven patients met these criteria in the study). Exhaled hydrogen and methane concentrations were measured using a BGA2000D (Laboratory for Expiration Biochemistry Nourishment Metabolism Co., Ltd., Nara, Japan).

According to the North American consensus [13], the following two diagnostic criteria for SIBO were used: a rise of ≥20 ppm from baseline in hydrogen by 90 min and a level of ≥10 ppm in methane. Furthermore, cases with elevated hydrogen levels were defined as H-SIBO, while those with elevated methane levels were defined as M-SIBO as previously reported [15]. M-SIBO was synonymous with IMO. In the study, both hydrogen- and methane-positive cases were included in the positive group of each subanalysis.

### Diagnosis of covert HE

The number connection test (NCT) is a neuropsychiatric test that entails using an iPad to connect the displayed numbers and alphabets with the fingers for the diagnosis of covert HE. In this study, we used “Neuro-Psychological Tests (v2.1) For iPad” available on the website (https://www.jsh.or.jp/medical/guidelines/medicalinfo/otsuka.html) provided by Otsuka Pharmaceutical Co., Ltd., and iPad mini 4 (Apple Inc., Cupertino, CA, USA) as the study device. Patients carried out the NCT-A and NCT-B tests, and covert HE was diagnosed on the basis of positive results of both NCT tests [16, 17]. The cutoff values were based on a previous report for Japanese patients [18].

### Observational study with rifaximin administration

In the 11 patients with covert HE and SIBO, we offered the opportunity to participate in the observational part of the study with rifaximin administration, and 8 patients agreed and were included. One to three months after the start of 1,200 mg/day rifaximin administration, laboratory tests, NCT, and breath test were reperformed in these patients.

### Statistical analysis

The Kolmogorov–Smirnov test was used to assess the normality of the distribution of continuous variables. The Mann–Whitney U and Pearson’s chi-squared tests were utilized to compare data of each cluster. The SPSS Statistics software (version 22.0; IBM, Armonk, NY, USA) was used to perform the Kolmogorov–Smirnov test, the Mann–Whitney U test, and Pearson’s chi-squared test.

## Results

The study cohort included 81 male and 26 female patients (n = 107), with a median age of 70 (40–86) years. Thirty-one patients (29.0%) were diagnosed with SIBO using the breath test, and we performed cluster analysis classified into two groups based on the existence of SIBO. In 77 patients with HCC complication, 64 patients were treated by transcatheter arterial embolization and/or chemotherapy, nine patients by systemic chemotherapy, two patients by radiofrequency ablation, one patient by radiation therapy, and one patient by surgical operation after the assessment of SIBO and covert HE. Seven patients were already introduced to systemic chemotherapy, but no cases complicated SIBO. The comparison showed no significant differences in age, gender, etiology of the LC, and liver function between the two groups (Table 1).

**Table 1:** The patients’ backgrounds and non-parametric tests clustered by SIBO.

median (min—max)	SIBO	Without SIBO	Mann–Whitney U and Pearson’s chi-square tests
or n (%)	N = 31	N = 76	P value
**Age, years**	70 (40–86)	70 (41–86)	0.721
**Gender**			
Males	20 (64.5)	61 (80.3)	0.085
Females	11 (35.5)	15 (19.7)	
**Body mass index, kg/m** ^**2**^	24.8 (12.6–38.7)	24.9 (17.1–47.7)	0.636
**The etiology of liver cirrhosis**			0.522
Hepatitis B virus	5 (16.1)	15 (19.7)	
Hepatitis C virus	10 (32.3)	13 (17.1)	
Alcoholic liver disease	7 (22.6)	24 (31.6)	
Non-alcoholic steatohepatitis	6 (19.4)	17 (22.4)	
Others	3 (9.7)	7 (9.2)	
**PPI administration**	16 (51.6)	47 (61.8)	0.329
**HCC complication**	21 (67.7)	56 (73.7)	0.535
With systemic chemotherapy	0 (0.0)	7 (9.2)	0.080
**Aspartate aminotransferase, U/L**	32 (17–209)	37 (12–146)	0.516
**Alanine aminotransferase, U/L**	25 (10–217)	30 (11–106)	0.408
**Alkaline Phosphatase, U/L**	247 (140–3147)	294 (64–1803)	0.278
**Gamma-glutamyl transpeptidase, U/L**	63 (16–341)	75 (13–691)	0.263
**Cholinesterase, U/L**	200 (82–341)	211 (47–484)	0.606
**Albumin, g/dL**	3.8 (2.1–4.5)	3.8 (2.3–5.0)	0.639
**Total bilirubin, mg/dL**	0.9 (0.5–6.2)	0.9 (0.3–14.2)	0.901
**Prothrombin time, %**	87 (25–131)	92 (39–124)	0.877
**Ammonia, μg/dL**	64 (28–242)	68 (30–184)	0.253
**Creatinine, mg/dL**	0.82 (0.49–1.98)	0.78 (0.45–3.31)	0.829
**Blood urea nitrogen, mg/dL**	17 (5–28)	16 (6–61)	0.351
**White blood cell count, x10** ^**3**^ **/μL**	4.3 (2.1–8.5)	4.5 (1.3–14.8)	0.530
**Platelet count, x10** ^**4**^ **/μL**	9.8 (4.9–22.8)	10.9 (2.6–26.3)	0.452
**Child-Pugh score**	5 (5–12)	5 (5–11)	0.493
Child Pugh grade (A/B/C)	21 / 8 / 2	64 / 9 / 3	0.153
**ALBI score**	-2.45 (-0.70–-3.12)	-2.44 (-0.47–-3.32)	0.616
mALBI grade (1/2a/2b/3)	10 / 7 / 11 / 3	28 / 22 / 20 / 6	0.757
**Covert HE**	11 (35.5)	19 (25.0)	0.273
NCT-A, s	50.3 (23.0–120.0)	50.2 (17.3–120.0)	0.424
NCT-B, s	109.3 (34.9–180.0)	91.9 (31.4–180.0)	0.132

SIBO, small intestinal bacterial overgrowth; PPI, proton pump inhibitor; HCC, hepatocellular carcinoma; ALBI, Albumin-Bilirubin; mALBI, modified ALBI; HE, hepatic encephalopathy; NCT, number connection test; *: P value < 0.05.

Next, we focused on the difference between H-SIBO and M-SIBO and performed reclassification into H-SIBO (16 cases, 15.0%) and others (91 cases, 85.0%). As a result of the comparison, the H-SIBO group had a significantly lower rate of HCC complication (50.0% vs. 75.8%, P = 0.034), a higher median Child-Pugh score (7 vs. 5, P = 0.037), and a higher rate of covert HE (50.0% vs. 24.2%, P = 0.034) (Table 2). In addition, univariate logistic analysis for the comparison between the two groups was performed. The results showed that the rate of HCC complication (P = 0.040), the median Child-Pugh score (P = 0.028), and the rate of covert HE (P = 0.040) were significantly different (Table 3). The Child-Pugh score was only derived in the multivariate logistic analysis (P = 0.028, odds ratio 1.39, 95% confidence interval 1.04–1.85). As a result of these analyses, the rate of H-SIBO was significantly associated with the patient’s liver function.

**Table 2:** Non-parametric tests clustered by H-SIBO.

median (min—max)	H-SIBO	Without H-SIBO	Mann–Whitney U and Pearson’s chi-square tests
or n (%)	N = 16	N = 91	P value
**Age, years**	72 (40–86)	69 (41–86)	0.428
**Gender**			
Males	10 (62.5)	71 (78.0)	0.182
Females	6 (37.5)	20 (22.0)	
**Body mass index, kg/m** ^**2**^	24.7 (12.6–34.7)	24.9 (17.1–47.7)	0.733
**The etiology of liver cirrhosis**			0.362
Hepatitis B virus	1 (6.3)	19 (20.9)	
Hepatitis C virus	5 (31.3)	18 (19.8)	
Alcoholic liver disease	4 (25.0)	27 (29.7)	
Non-alcoholic steatohepatitis	3 (18.8)	20 (22.0)	
Others	3 (18.8)	7 (7.7)	
**PPI administration**	10 (62.5)	53 (58.2)	0.750
**HCC complication**	8 (50.0)	69 (75.8)	**0.034** *
With systemic chemotherapy	0 (0.0)	7 (9.2)	0.251
**Aspartate aminotransferase, U/L**	29 (17–209)	37 (12–146)	0.474
**Alanine aminotransferase, U/L**	22 (10–217)	30 (11–106)	0.152
**Alkaline Phosphatase, U/L**	224 (151–3147)	293 (64–1803)	0.299
**Gamma-glutamyl transpeptidase, U/L**	51 (16–341)	73 (13–691)	0.166
**Cholinesterase, U/L**	183 (82–316)	211 (47–484)	0.297
**Albumin, g/dL**	3.7 (2.1–4.2)	3.8 (2.3–5.0)	0.220
**Total bilirubin, mg/dL**	0.9 (0.5–6.2)	0.9 (0.3–14.2)	0.799
**Prothrombin time, %**	81 (25–115)	92 (39–131)	0.330
**Ammonia, μg/dL**	64 (28–242)	65 (30–184)	0.204
**Creatinine, mg/dL**	0.85 (0.52–1.98)	0.79 (0.45–3.31)	0.714
**Blood urea nitrogen, mg/dL**	18 (5–28)	16 (6–61)	0.250
**White blood cell count, x10** ^**3**^ **/μL**	4.3 (2.1–8.5)	4.3 (1.3–14.8)	0.471
**Platelet count, x10** ^**4**^ **/μL**	11.6 (4.9–22.8)	10.8 (2.6–26.3)	0.965
**Child-Pugh score**	7 (5–12)	5 (5–11)	**0.037** *
Child Pugh grade (A/B/C)	8 / 6 / 2	77 / 11 / 3	**0.007** *
**ALBI score**	-2.31 (-0.70–-2.87)	-2.45 (-0.47–-3.32)	0.205
mALBI grade (1/2a/2b/3)	5 / 3 / 5 / 3	33 / 26 / 26 / 6	0.391
**Covert HE**	8 (50.0)	22 (24.2)	**0.034** *
NCT-A, s	55.2 (23.0–120.0)	49.9 (17.3–120.0)	0.151
NCT-B, s	111.2 (34.9–180.0)	91.6 (31.4–180.0)	0.055

H-SIBO, hydrogen producing small intestinal bacterial overgrowth; PPI, proton pump inhibitor; HCC, hepatocellular carcinoma; ALBI, Albumin-Bilirubin; mALBI, modified ALBI; HE, hepatic encephalopathy; NCT, number connection test;

*: P value < 0.05.

**Table 3:** Univariate and multivariate logistic analyses clustered by H-SIBO.

median (min—max)	Univariable logistic regression		Multivariable logistic regression	
or n (%)	Odds ratio (95% CI)	P value	Odds ratio (95% CI)	P value
**Age, years**	1.00 (0.95–1.05)	0.969		
**Gender**	0.47 (0.15–1.45)	0.189		
**Body mass index, kg/m** ^**2**^	0.96 (0.86–1.07)	0.455		
**PPI administration**	1.20 (0.40–3.57)	0.750		
**HCC complication**	**0.32 (0.11–0.95)**	**0.040** *		0.146
**Aspartate aminotransferase, U/L**	1.00 (0.99–1.02)	0.560		
**Alanine aminotransferase, U/L**	1.00 (0.99–1.02)	0.727		
**Alkaline Phosphatase, U/L**	1.00(1.00–1.00)	0.303		
**Gamma-glutamyl transpeptidase, U/L**	1.00 (0.99–1.00)	0.312		
**Cholinesterase, U/L**	1.00 (0.99–1.00)	0.255		
**Albumin, g/dL**	0.44 (0.18–1.09)	0.075		
**Total bilirubin, mg/dL**	1.10 (0.83–1.45)	0.511		
**Prothrombin time, %**	0.98 (0.95–1.00)	0.065		
**Ammonia, μg/dL**	1.00 (0.98–1.02)	0.952		
**Creatinine, mg/dL**	1.42 (0.36–5.68)	0.617		
**Blood urea nitrogen, mg/dL**	1.02 (0.96–1.09)	0.559		
**White blood cell count, x10** ^**3**^ **/μL**	1.00 (1.00–1.00)	0.415		
**Platelet count, x10** ^**4**^ **/μL**	0.99 (0.90–1.09)	0.865		
**Child-Pugh score**	**1.39 (1.04–1.85)**	**0.028** *	**1.39 (1.04–1.85)**	**0.028** *
**ALBI score**	2.15 (0.89–5.16)	0.087		
**Covert HE**	**3.14 (1.05–9.34)**	**0.040** *		0.101
NCT-A, s	1.02 (1.00–1.04)	0.092		
NCT-B, s	1.01 (1.00–1.02)	0.087		

H-SIBO, hydrogen producing small intestinal bacterial overgrowth; CI, confidence interval; PPI, proton pump inhibitor; HCC, hepatocellular carcinoma; ALBI, Albumin-Bilirubin; mALBI, modified ALBI; HE, hepatic encephalopathy; NCT, number connection test;

*: P value < 0.05.

Furthermore, there were no significant factors without the administration rate of proton pump inhibitors (36.8% vs. 63.6%, P = 0.031) in the comparison after the reclassification into M-SIBO/IMO (19 cases, 17.8%) and others (88 cases, 82.2%) in the same method as hydrogen (Table 4).

**Table 4:** Non-parametric tests clustered by M-SIBO / IMO.

median (min—max)	M-SIBO	Without M-SIBO	Mann–Whitney U and Pearson’s chi-square tests
or n (%)	N = 19	N = 88	P value
**Age, years**	69 (53–86)	70 (40–86)	0.987
**Gender**			
Males	13 (68.4)	68 (77.3)	0.415
Females	6 (31.6)	20 (22.7)	
**Body mass index, kg/m** ^**2**^	24.8 (17.7–38.7)	24.9 (12.6–47.7)	0.967
**The etiology of liver cirrhosis**			0.271
Hepatitis B virus	4 (21.1)	16 (18.2)	
Hepatitis C virus	7 (36.8)	16 (18.2)	
Alcoholic liver disease	5 (26.3)	26 (29.5)	
Non-alcoholic steatohepatitis	3 (15.8)	20 (22.7)	
Others	0 (0.0)	10 (11.4)	
**PPI administration**	7 (36.8)	56 (63.6)	**0.031** *
**HCC complication**	16 (84.2)	61 (69.3)	0.190
With systemic chemotherapy	0 (0.0)	7 (9.2)	0.203
**Aspartate aminotransferase, U/L**	45 (21–92)	36 (12–209)	0.392
**Alanine aminotransferase, U/L**	30 (16–83)	29 (10–217)	0.579
**Alkaline Phosphatase, U/L**	281 (140–444)	284 (64–3147)	0.823
**Gamma-glutamyl transpeptidase, U/L**	65 (24–194)	69 (13–691)	0.912
**Cholinesterase, U/L**	200 (125–341)	211 (47–484)	0.944
**Albumin, g/dL**	3.8 (3.0–4.5)	3.8 (2.1–5.0)	0.731
**Total bilirubin, mg/dL**	0.9 (0.5–1.9)	0.9 (0.3–14.2)	0.860
**Prothrombin time, %**	87 (62–131)	92 (25–124)	0.723
**Ammonia, μg/dL**	64 (30–242)	66 (28–184)	0.913
**Creatinine, mg/dL**	0.82 (0.49–1.33)	0.78 (0.45–3.31)	0.906
**Blood urea nitrogen, mg/dL**	17 (9–26)	16 (5–61)	0.662
**White blood cell count, x10** ^**3**^ **/μL**	4.3 (2.1–6.9)	4.5 (1.3–14.8)	0.577
**Platelet count, x10** ^**4**^ **/μL**	9.8 (7.4–19.1)	10.9 (2.6–26.3)	0.517
**Child-Pugh score**	5 (5–8)	5 (5–12)	0.384
Child Pugh grade (A/B/C)	15 / 4 / 0	70 / 13 / 5	0.480
**ALBI score**	-2.45 (-1.57–-3.12)	-2.44 (-0.47–-3.32)	0.711
mALBI grade (1/2a/2b/3)	6 / 5 / 8 / 0	32 / 24 / 23 / 9	0.331
**Covert HE**	6 (31.6)	24 (27.3)	0.705
NCT-A, s	50.3 (24.6–120.0)	50.2 (17.3–120.0)	0.681
NCT-B, s	118.2 (41.0–180.0)	92.5 (31.4–180.0)	0.239

M-SIBO, methane producing small intestinal bacterial overgrowth; IMO, intestinal methanogen overgrowth; PPI, proton pump inhibitor; HCC, hepatocellular carcinoma; ALBI, Albumin-Bilirubin; mALBI, modified ALBI; HE, hepatic encephalopathy; NCT, number connection test;

*: P value < 0.05.

Additionary, the direct comparison of M-SIBO and H-SIBO did not significantly differ, because the case number was small in both groups (S1 Table).

**Fig 2:**
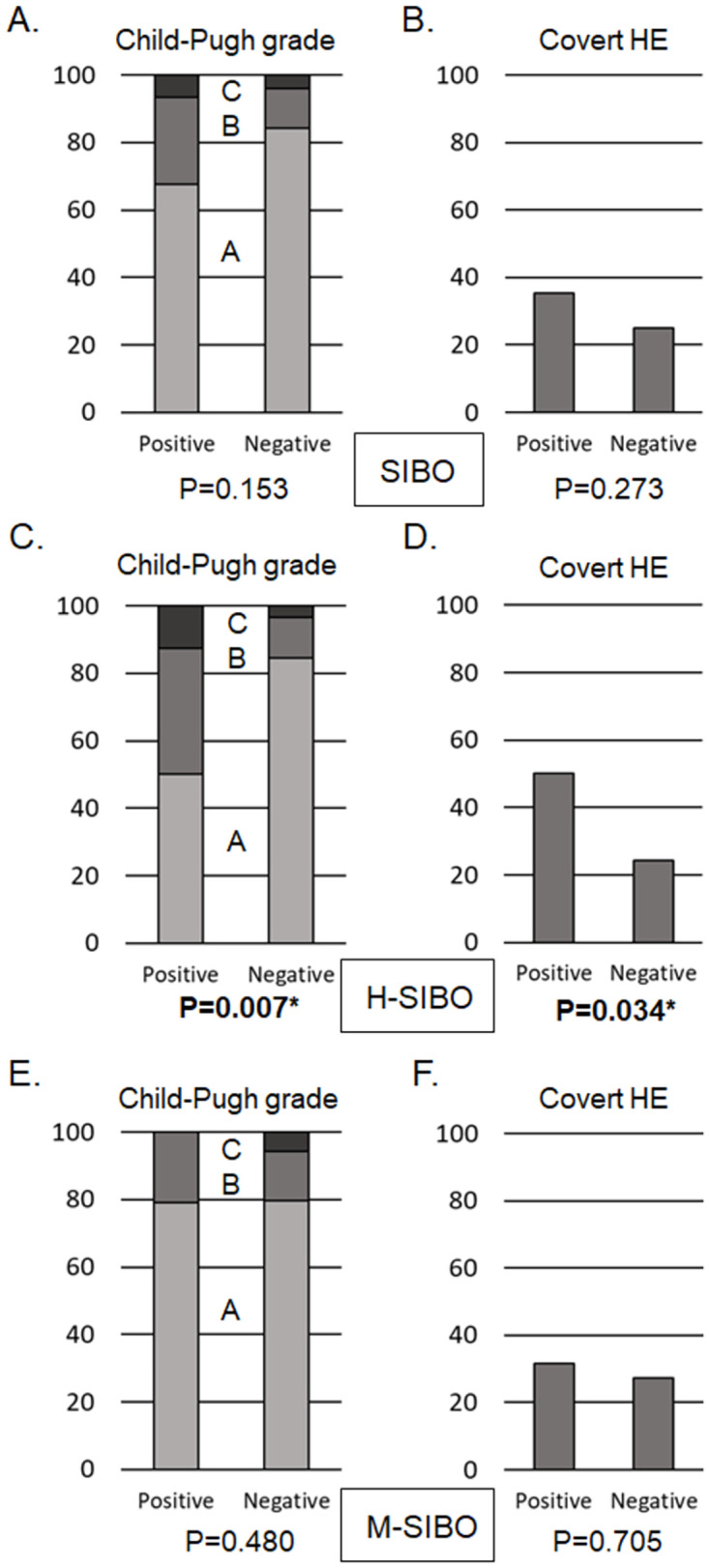
Relationship between the child-pugh scores of covert HE and the type of small intestinal bacterial overgrowth (SIBO). Overall, SIBO was not significantly associated with the Child-Pugh grade (grade A of 67.7%, grade B of 25.8%, and grade C of 6.5% in SIBO positive and grade A of 84.2%, grade B of 11.8%, and grade C of 3.9% in SIBO negative, P = 0.153; A) or covert hepatic encephalopathy (HE) (35.5% vs. 25.0%, P = 0.273; B). By contrast, hydrogen-producing small intestinal bacterial overgrowth (H-SIBO) was significantly associated with both Child-Pugh grade (grade A of 50.0%, grade B of 37.5%, and grade C of 12.5% in H-SIBO positive and grade A of 84.6%, grade B of 12.1%, and grade C of 3.3% in H-SIBO negative, P = 0.007; C) and covert HE (50.0% vs. 24.2%, P = 0.034; D). In addition, methane-producing small intestinal bacterial overgrowth (M-SIBO) was also not significantly associated with the Child-Pugh grade (grade A of 78.9%, grade B of 21.1%, and grade C of 0.0% in M-SIBO positive and grade A of 79.5%, grade B of 14.8%, and grade C of 5.7% in M-SIBO negative, P = 0.480; E), or covert HE (31.6% vs. 27.3%, P = 0.705; F).

Fig 2 summarizes the relationship between liver function and the type of SIBO. Overall SIBO has no significant association with the Child-Pugh grade (grade A of 67.7%, grade B of 25.8%, and grade C of 6.5% in SIBO positive and grade A of 84.2%, grade B of 11.8%, and grade C of 3.9% in SIBO negative, P = 0.153; Fig 2A) or covert HE (35.5% vs. 25.0%, P = 0.273; Fig 2B). On the contrary, H-SIBO was significantly associated with both the Child-Pugh grade (grade A of 50.0%, grade B of 37.5%, and grade C of 12.5% in H-SIBO positive and grade A of 84.6%, grade B of 12.1%, and grade C of 3.3% in H-SIBO negative, P = 0.007; Fig 2C) and covert HE (50.0% vs. 24.2%, P = 0.034; Fig 2D). In addition, there was no significant association between M-SIBO/IMO and the Child-Pugh grade (grade A of 78.9%, grade B of 21.1%, and grade C of 0.0% in M-SIBO positive and grade A of 79.5%, grade B of 14.8%, and grade C of 5.7% in M-SIBO negative, P = 0.480; Fig 2E) or covert HE (31.6% vs. 27.3%, P = 0.705; Fig 2F).

In addition, we evaluated the therapeutic response of SIBO in eight patients with covert HE with SIBO (five cases of M-SIBO/IMO and three cases of H-SIBO) treated with the oral administration of 1,200 mg/day rifaximin. Only one of the five (20%) patients with M-SIBO/IMO and two of the three (67%) patients with H-SIBO showed an improvement of the breath test. Furthermore, none of the five (0%) patients with M-SIBO/IMO and one of the two (50%) patients with H-SIBO showed an improvement of the score of NCT under the diagnostic borderline of covert HE (Table 5). One patient with H-SIBO was excluded since the patient had grade 1 HE at the time of pre-treatment of the breath test and did not undergo the NCT.

**Table 5:** Case series treated by rifaximin in patients with both SIBO and covert HE.

Case number	1	2	3	4	5	6	7	8
SIBO type	Methane	Methane	Methane	Methane	Methane	Hydrogen	Hydrogen	Hydrogen
**Age (year)**	73	65	55	61	69	47	66	70
**Gender**	M	M	F	F	M	M	M	M
**Child-Pugh Score**	5	5	6	7	5	12	11	8
**Child-Pugh grade**	A	A	A	B	A	C	C	B
**Breath hydrogen concentration** (ppm, pre to post)	10 to 5	15 to 5	10 to 7	10 to 5	17 to 8	31 to 41	57 to 7	36 to 3
**Breath methane concentration** (ppm, pre to post)	18 to 2	17 to 46	63 to 37	30 to 37	11 to 26	3 to 2	2 to 1	4 to 0
**SIBO improve**	**Yes**	No	No	No	No	No	**Yes**	**Yes**
**NCT-A** (sec, pre to post)	50 to 109	34 to 49	46 to 71	31 to 53	64 to 73	64 to 68	N.A. to 66	120 to 84
**NCT-B** (sec, pre to post)	180 to 180	41 to 65	180 to 93	56 to 82	166 to 180	75 to 79	N.A. to 98	180 to 134
**Serum ammonia level** (μg/dL, pre to post)	36 to 67	68 to 52	N.A.	112 to 93	93 to 118	151 to 223	123 to 90	70 to 53
**HE improve**	No	No	No	No	No	No	N.A.	**Yes**

SIBO, small intestinal bacterial overgrowth; M, male; F, female; NCT, number connection test; HE, hepatic encephalopathy; N.A., not available.

## Discussion

Although the diagnostic method of SIBO by breath test measurement of methane and hydrogen has been reported since over 20 years ago [19], only the hydrogen-producing type SIBO was measured and judged to be SIBO in the previously mentioned meta-analysis for LC [12] in our investigation. There was no significant relationship between SIBO and liver function and HE in the study because we included the methane-producing type in the diagnosis of SIBO. Previous studies measuring only hydrogen levels using the breath test showed the same analyses and results as those in Table 2 in the study. On the other hand, it was shown that the methane-producing type is not associated with liver function in the study. In the field of IBS, H-SIBO has been associated with diarrhea-predominant type or mixed bowel habits type of IBS, while M-SIBO/IMO has been associated with constipation-predominant type of IBS [20]. Furthermore, in a report that assessed the relationship between SIBO and severe heart failure, only M-SIBO/IMO was significantly associated with the event-free survival of heart failure [21]; therefore, these findings suggesting that the pathological significance of the high level of hydrogen or methane in exhaled air as measured using the breath test were also different in liver diseases. In the results of the study, M-SIBO was not significantly associated with both liver function and HE, suggesting that the pathological significance of M-SIBO is unknown for LC, unlike that of H-SIBO.

While there are some methods for diagnosing covert HE, neuropsychological tests are commonly used because it has good external validity [22, 23]. However, there are no standardized for diagnosing covert HE using neuropsychological tests, and psychometric hepatic encephalopathy score (PHES) is widely used for this purpose. PHES included seven tests: the line tracing test, the serial dotting test, the digit symbol test, NCTs A and B, the digit span test and the canceling d-test [24]. Because PHES requires many tests and patient burden, that takes about 30 min in some cases [22]. The NCTs were widely used as psychometric tests in cirrhotic patients. Also, several reports have used small amount of neuropsychological tests than PHES to diagnose covert HE, furthermore to increase the specificity, the positive of two or more tests are considered covert HE [16, 17]. Therefore, in this study, covert HE was diagnosed both NCT A and B positive.

PPIs are associated with SIBO development [25], and in liver disease, PPIs are also associated with HE [26], which is thought to cause intestinal dysbiosis. However, there was no association between SIBO and PPI in this study. Although there was no significance, PPI use was higher in H-SIBO, so there may be an association with the PPI use.

According to the practice guidelines of the American Association for the Study of Liver Diseases and the European Association for the Study of the Liver [22] and the Japanese Society of Gastroenterology and the Japan Society of Hepatology [27], lactulose is the first-choice treatment for overt HE, and rifaximin is an add-on therapy to lactulose for the prevention of overt HE recurrence. Although many studies have reported the usefulness of poorly absorbable oral antibiotics for HE [28–30], the pharmacological mechanism of rifaximin is now a subject of debate. Not only antibacterial effects but also the function of tight junctions has been reported the efficiency of rifaximin [31]. It is also known that the rifaximin does not alter the intestinal microbiota in the stool, in spite of its efficiency [32], and this result is similar to ours. The fecal microbiotas do not necessarily reflect that of the small intestine; therefore, the breath test may be effective for the assessment and prediction of efficiency. The present study suggests that H-SIBO, but not M-SIBO, may be responsive to rifaximin. In this study, only eight patients were evaluated, and further studies were needed. However, with a small number of cases similar to that in our study, it has been reported that H-SIBO is more responsive to rifaximin treatment than M-SIBO [33]. Furthermore, in the field of IBS, M-SIBO is resistant to antibiotics, which is consistent with our results [34]. Therefore, it is assumed that rifaximin treatment for covert HE with M-SIBO is associated with a poor response and is of little significance because M-SIBO is antibiotic resistant and rarely associated with liver function.

The standard treatment for SIBO is poorly absorbable oral antibiotics and the reduction of fermentable products in the diet. However, prebiotics such as lactulose should also be avoided because they could lead to an increase in fermentable products [14]. Therefore, HE without SIBO was treated with lactulose and/or rifaximin. HE with H-SIBO should be mainly treated with poorly absorbable oral antibiotics; however, M-SIBO requires further investigation.

## Conclusions

H-SIBO was significantly associated with liver function although there was no independent correlation between covert HE and both H-SIBO and M-SIBO. Furthermore, it was suggested that rifaximin might be more effective for covert HE with H-SIBO than with M-SIBO. Therefore, the diagnosis of SIBO, including the classification as H-SIBO and M-SIBO using the breath test, could help to determine the choice of treatment for HE.

## Supporting information

S1 Table The comparison between H-SIBO and M-SIBO. (DOCX)

## References

[1] D’Amico G, Garcia-Tsao G, Pagliaro L. Natural history and prognostic indicators of survival in cirrhosis: a systematic review of 118 studies. J Hepatol. 2006; 44(1): 217–231. doi: 10.1016/j.jhep.2005.10.013 16298014

[2] Weissenborn K. Hepatic Encephalopathy: Definition, Clinical Grading and Diagnostic Principles. Drugs. 2019;79(Suppl 1):5–9. doi: 10.1007/s40265-018-1018-z 30706420 PMC6416238

[3] Bustamante J, Rimola A, Ventura PJ, Navasa M, Cirera I, Reggiardo V, et al. Prognostic significance of hepatic encephalopathy in patients with cirrhosis. J Hepatol. 1999;30(5):890–5. doi: 10.1016/s0168-8278(99)80144-5 10365817

[4] Wijdicks EF. Hepatic Encephalopathy. N Engl J Med. 2016;375(17):1660–70. doi: 10.1056/NEJMra1600561 27783916

[5] Saunders JB, Walters JR, Davies AP, Paton A. A 20-year prospective study of cirrhosis. Br Med J (Clin Res Ed). 1981;282(6260):263–6. doi: 10.1136/bmj.282.6260.263 6779978 PMC1504019

[6] Romero-Gómez M, Boza F, García-Valdecasas MS, García E, Aguilar-Reina J. Subclinical hepatic encephalopathy predicts the development of overt hepatic encephalopathy. Am J Gastroenterol. 2001;96(9):2718–23. doi: 10.1111/j.1572-0241.2001.04130.x 11569701

[7] Kato A, Watanabe Y, Sawara K, Suzuki K. Diagnosis of sub-clinical hepatic encephalopathy by Neuropsychological Tests (NP-tests). Hepatol Res. 2008;38 Suppl 1:S122–7. doi: 10.1111/j.1872-034X.2008.00437.x 19125943

[8] Wang JY, Bajaj JS, Wang JB, Shang J, Zhou XM, Guo XL, et al. Lactulose improves cognition, quality of life, and gut microbiota in minimal hepatic encephalopathy: A multicenter, randomized controlled trial. J Dig Dis. 2019;20(10):547–56. doi: 10.1111/1751-2980.12816 31448533

[9] Wang AJ, Peng AP, Li BM, Gan N, Pei L, Zheng XL, et al. Natural history of covert hepatic encephalopathy: An observational study of 366 cirrhotic patients. World J Gastroenterol. 2017;23(34):6321–9. doi: 10.3748/wjg.v23.i34.6321 28974899 PMC5603499

[10] Ghosh G, Jesudian AB. Small Intestinal Bacterial Overgrowth in Patients With Cirrhosis. J Clin Exp Hepatol. 2019;9(2):257–67. doi: 10.1016/j.jceh.2018.08.006 31024208 PMC6477138

[11] Choung RS, Ruff KC, Malhotra A, Herrick L, Locke GR 3rd, Harmsen WS, et al. Clinical predictors of small intestinal bacterial overgrowth by duodenal aspirate culture. Aliment Pharmacol Ther. 2011;33(9):1059–67. doi: 10.1111/j.1365-2036.2011.04625.x 21395630

[12] Maslennikov R, Pavlov C, Ivashkin V. Small intestinal bacterial overgrowth in cirrhosis: systematic review and meta-analysis. Hepatol Int. 2018;12(6):567–76. doi: 10.1007/s12072-018-9898-2 30284684

[13] Quigley EM, Abu-Shanab A. Small intestinal bacterial overgrowth. Infect Dis Clin North Am. 2010;24(4):943–59, viii–ix. doi: 10.1016/j.idc.2010.07.007 20937459

[14] Pimentel M, Saad RJ, Long MD, Rao SSC. ACG Clinical Guideline: Small Intestinal Bacterial Overgrowth. Am J Gastroenterol. 2020;115(2):165–78. doi: 10.14309/ajg.0000000000000501 32023228

[15] Suri J, Kataria R, Malik Z, Parkman HP, Schey R. Elevated methane levels in small intestinal bacterial overgrowth suggests delayed small bowel and colonic transit. Medicine (Baltimore). 2018;97(21):e10554. doi: 10.1097/MD.0000000000010554 29794732 PMC6393144

[16] Kato A, Tanaka H, Kawaguchi T, Kanazawa H, Iwasa M, Sakaida I, et al. Nutritional management contributes to improvement in minimal hepatic encephalopathy and quality of life in patients with liver cirrhosis: A preliminary, prospective, open-label study. Hepatol Res. 2013;43(5):452–8. doi: 10.1111/j.1872-034X.2012.01092.x 22994429

[17] Adrover R, Barrio M, Malca Albuquerque M, Romé J, Borzi S, Cocozzella D, et al. Validation of the number connection test for identifying patients with minimal hepatic encephalopathy. Acta Gastroenterol Latinoam. 2012;42(2):105–11. 22876712

[18] Kawaguchi T, Konishi M, Kato A, Kato M, Kooka Y, Sawara K, et al. Updating the neuropsychological test system in Japan for the elderly and in a modern touch screen tablet society by resetting the cut-off values. Hepatol Res. 2017;47(12):1335–9. doi: 10.1111/hepr.12864 28066966

[19] Yang CY, Chang CS, Chen GH. Small-intestinal bacterial overgrowth in patients with liver cirrhosis, diagnosed with glucose H2 or CH4 breath tests. Scand J Gastroenterol. 1998;33(8):867–71. doi: 10.1080/00365529850171549 9754736

[20] Takakura W, Pimentel M. Small Intestinal Bacterial Overgrowth and Irritable Bowel Syndrome—An Update. Front Psychiatry. 2020;11:664. doi: 10.3389/fpsyt.2020.00664 32754068 PMC7366247

[21] Song Y, Liu Y, Qi B, Cui X, Dong X, Wang Y, et al. Association of Small Intestinal Bacterial Overgrowth With Heart Failure and Its Prediction for Short-Term Outcomes. J Am Heart Assoc. 2021;10(7):e015292. doi: 10.1161/JAHA.119.015292 33728933 PMC8174348

[22] Vilstrup H, Amodio P, Bajaj J, Cordoba J, Ferenci P, Mullen KD, et al. Hepatic encephalopathy in chronic liver disease: 2014 Practice Guideline by the American Association for the Study of Liver Diseases and the European Association for the Study of the Liver. Hepatology. 2014;60(2):715–35. doi: 10.1002/hep.27210 25042402

[23] Weissenborn K, Ennen JC, Schomerus H, Rückert N, Hecker H. Neuropsychological characterization of hepatic encephalopathy. J Hepatol. 2001;34(5):768–73. doi: 10.1016/s0168-8278(01)00026-5 11434627

[24] Schomerus H, Hamster W. Neuropsychological aspects of portal-systemic encephalopathy. Metab Brain Dis. 1998;13(4):361–77. doi: 10.1023/a:1020645110880 10206827

[25] Vaezi MF, Yang YX, Howden CW. Complications of Proton Pump Inhibitor Therapy. Gastroenterology. 2017;153(1):35–48. doi: 10.1053/j.gastro.2017.04.047 28528705

[26] Fasullo M, Rau P, Liu DQ, Holzwanger E, Mathew JP, Guilarte-Walker Y, et al. Proton pump inhibitors increase the severity of hepatic encephalopathy in cirrhotic patients. World J Hepatol. 2019;11(6):522–30. doi: 10.4254/wjh.v11.i6.522 31293720 PMC6603505

[27] Yoshiji H, Nagoshi S, Akahane T, Asaoka Y, Ueno Y, Ogawa K, et al. Evidence-based clinical practice guidelines for Liver Cirrhosis 2020. J Gastroenterol. 2021;56(7):593–619. doi: 10.1007/s00535-021-01788-x 34231046 PMC8280040

[28] Bass NM, Mullen KD, Sanyal A, Poordad F, Neff G, Leevy CB, et al. Rifaximin treatment in hepatic encephalopathy. N Engl J Med. 2010;362(12):1071–81. doi: 10.1056/NEJMoa0907893 20335583

[29] Kang SH, Lee YB, Lee JH, Nam JY, Chang Y, Cho H, et al. Rifaximin treatment is associated with reduced risk of cirrhotic complications and prolonged overall survival in patients experiencing hepatic encephalopathy. Aliment Pharmacol Ther. 2017;46(9):845–55. doi: 10.1111/apt.14275 28836723

[30] Lighthouse J, Naito Y, Helmy A, Hotten P, Fuji H, Min CH, et al. Endotoxinemia and benzodiazepine-like substances in compensated cirrhotic patients: a randomized study comparing the effect of rifaximine alone and in association with a symbiotic preparation. Hepatol Res. 2004;28(3):155–60. doi: 10.1016/j.hepres.2003.11.005 15036072

[31] Douhara A, Moriya K, Yoshiji H, Noguchi R, Namisaki T, Kitade M, et al. Reduction of endotoxin attenuates liver fibrosis through suppression of hepatic stellate cell activation and remission of intestinal permeability in a rat non-alcoholic steatohepatitis model. Mol Med Rep. 2015;11(3):1693–700. doi: 10.3892/mmr.2014.2995 25421042 PMC4270343

[32] Kaji K, Takaya H, Saikawa S, Furukawa M, Sato S, Kawaratani H, et al. Rifaximin ameliorates hepatic encephalopathy and endotoxemia without affecting the gut microbiome diversity. World J Gastroenterol. 2017;23(47):8355–66. doi: 10.3748/wjg.v23.i47.8355 29307995 PMC5743506

[33] Barkin JA, Keihanian T, Barkin JS, Antequera CM, Moshiree B. Preferential usage of rifaximin for the treatment of hydrogen-positive smallintestinal bacterial overgrowth. Rev Gastroenterol Peru. 2019;39(2):111–5. 31333225

[34] Pimentel M, Chang C, Chua KS, Mirocha J, DiBaise J, Rao S, et al. Antibiotic treatment of constipation-predominant irritable bowel syndrome. Dig Dis Sci. 2014;59(6):1278–85. doi: 10.1007/s10620-014-3157-8 24788320

