# Supplementary material for: Hydrogen-producing small intestinal bacterial overgrowth is associated with hepatic encephalopathy and liver function
Source: PLoS One. 2022 Feb 25;17(2):e0264459. doi: 10.1371/journal.pone.0264459 (PMC8880851; doi:10.1371/journal.pone.0264459)
Supplement: S1 Table — (DOCX) [file pone.0264459.s001.docx]

**S1 Table. The comparison between H-SIBO and M-SIBO.**

| median (min – max) | **H-SIBO** | **M-SIBO** | Mann–Whitney U and　Pearson's chi-square tests |
| --- | --- | --- | --- |
| or　n (%) | N = 16 | N = 19 | **P value** |
| **Age, years** | 72 (40 - 86) | 69 (53 - 86) | 0.589 |
| **Gender** |  |  |  |
| Males | 10 (62.5) | 13 (68.4) | 0.736 |
| Females | 6 (37.5) | 6 (31.6) |  |
| **Body mass index, kg/m^2^** | 24.7 (12.6 - 34.7) | 24.8 (17.7 - 38.7) | 0.756 |
| **The etiology of liver cirrhosis**  Hepatitis B virus  Hepatitis C virus  Alcoholic liver disease  Non-alcoholic steatohepatitis  Others | 1 ( 6.3)  5 (31.3)  4 (25.0)  3 (18.8)  3 (18.8) | 4 (21.1)  7 (36.8)  5 (26.3)  3 (15.8)  0 ( 0.0) | 0.330 |
| **PPI　administration** | 10 (62.5) | 7 (36.8) | 0.181 |
| **HCC complication** | 8 (50.0) | 16 (84.2) | 0.065 |
| **Aspartate aminotransferase, U/L** | 29 (17 - 209) | 45 (21 - 92) | 0.385 |
| **Alanine aminotransferase, U/L** | 22 (10 - 217) | 30 (16 - 83) | 0.133 |
| **Alkaline Phosphatase, U/L** | 224 (151 – 3147) | 281 (140 – 444) | 0.385 |
| **Gamma-glutamyl transpeptidase, U/L** | 51 (16 – 341) | 65 (24 – 194) | 0.257 |
| **Cholinesterase, U/L** | 183 (82 – 316) | 200 (125 – 341) | 0.441 |
| **Albumin, g/dL** | 3.7 (2.1 - 4.2) | 3.8 (3.0 - 4.5) | 0.317 |
| **Total bilirubin, mg/dL** | 0.9 (0.5 – 6.2) | 0.9 (0.5 – 1.9) | 0.935 |
| **Prothrombin time, %** | 81 (25 - 115) | 87 (62 - 131) | 0.367 |
| **Ammonia, μg/dL** | 64 (28 - 242) | 64 (30 - 242) | 0.605 |
| **Creatinine, mg/dL** | 0.85 (0.52 - 1.98) | 0.82 (0.49 - 1.33) | 0.635 |
| **Blood urea nitrogen, mg/dL** | 18 (5 – 28) | 17 (9 – 26) | 0.545 |
| **White blood cell count, x10^3^/µL** | 4.3 (2.1 – 8.5) | 4.3 (2.1 – 6.9) | 0.935 |
| **Platelet count, x10^4^/µL** | 11.6 (4.9 - 22.8) | 9.8 (7.4 - 19.1) | 0.635 |
| **Child-Pugh score** | 7 (5 – 12) | 5 (5 – 8) | 0.125 |
| Child Pugh grade (A/B/C) | 8 / 6 / 2 | 15 / 4 / 0 | 0.103 |
| **ALBI score** | -2.31 (-0.70 - -2.87) | -2.45 (-1.57 - -3.12) | 0.271 |
| mALBI grade (1/2a/2b/3) | 5 / 3 / 5 / 3 | 6 / 5 / 8 / 0 | 0.319 |
| **Covert HE** | 8 (50.0) | 6 (31.6) | 0.317 |
| NCT-A, s | 55.2 (23.0 – 120.0) | 50.3 (24.6 – 120.0) | 0.502 |
| NCT-B, s | 111.2 (34.9 – 180.0) | 118.2 (41.0 – 180.0) | 0.683 |
| H-SIBO, hydrogen producing small intestinal bacterial overgrowth; M-SIBO, methane producing small intestinal bacterial overgrowth; PPI, proton pump inhibitor; HCC, hepatocellular carcinoma; ALBI, Albumin-Bilirubin; mALBI, modified ALBI; HE, hepatic encephalopathy; NCT, number connection test; *: P value < 0.05. | | | |
